# Insights into the mechanism of X-ray-induced disulfide-bond cleavage in lysozyme crystals based on EPR, optical absorption and X-ray diffraction studies

**DOI:** 10.1107/S0907444913022117

**Published:** 2013-11-19

**Authors:** Kristin A. Sutton, Paul J. Black, Kermit R. Mercer, Elspeth F. Garman, Robin L. Owen, Edward H. Snell, William A. Bernhard

**Affiliations:** a Hauptman–Woodward Medical Research Institute, 700 Ellicott Street, Buffalo, NY 14086, USA; b University of Rochester Medical Center, Rochester, NY 14642, USA; c Laboratory of Molecular Biophysics, Department of Biochemistry, University of Oxford, South Parks Road, Oxford, Oxfordshire OX1 3QU, England; d Diamond Light Source, Harwell Science and Innovation Campus, Didcot, Oxfordshire OX11 0DE, England; e Department of Structural Biology, SUNY Buffalo Medical School, 700 Ellicott Street, Buffalo, NY 14203, USA

**Keywords:** radiation damage, protein, disulfide bonds, UV–visible absorption microspectrophotometry, electron paramagnetic resonance

## Abstract

Electron paramagnetic resonance (EPR) and online UV–visible absorption microspectrophotometry with X-ray crystallography have been used in a complementary manner to follow X-ray-induced disulfide-bond cleavage. Online UV–visible spectroscopy showed that upon X-irradiation, disulfide radicalization appeared to saturate at an absorbed dose of approximately 0.5–0.8 MGy, in contrast to the saturating dose of ∼0.2 MGy observed using EPR at much lower dose rates. The observations suggest that a multi-track model involving product formation owing to the interaction of two separate tracks is a valid model for radiation damage in protein crystals. The saturation levels are remarkably consistent given the widely different experimental parameters and the range of total absorbed doses studied. The results indicate that even at the lowest doses used for structural investigations disulfide bonds are already radicalized. Multi-track considerations offer the first step in a comprehensive model of radiation damage that could potentially lead to a combined computational and experimental approach to identifying when damage is likely to be present, to quantitate it and to provide the ability to recover the native unperturbed structure.

## Abstract 2

Electron paramagnetic resonance (EPR) and online UV–visible absorption microspectrophotometry with X-ray crystallography have been used in a complementary manner to follow X-ray-induced disulfide-bond cleavage, to confirm a multi-track radiation-damage process and to develop a model of that process.

## 1. Introduction   

Macromolecular X-ray crystallography subjects the crystal to typical X-ray doses of the order of kilograys (kGy) per image. Multiple images are used to build up a complete data set. To put this in perspective, the LD_50_ for a human (the dose for which 50% of the affected population do not survive) is 4.5 Gy (Mole, 1984 ▶). Cryoprotection techniques (Rodgers, 1997 ▶; Garman & Schneider, 1997 ▶) account for some of our ability to reduce the rate of radiation damage, as do the large number of repeating units within a crystal; however, it should be noted that structural effects owing to radiation damage are more likely to be present in crystals than not. Specific structural damage to particular covalent bonds occurs in a reproducible order. Firstly disulfide bridges elongate and then break, secondly glutamates and aspartates are decarboxylated, thirdly tyrosine residues lose their hydroxyl group and fourthly the carbon–sulfur bonds in methionines are cleaved (Weik *et al.*, 2000 ▶, 2002 ▶; Burmeister, 2000 ▶; Ravelli & McSweeney, 2000 ▶). These structural effects occur before global effects on the diffraction quality are observed, *i.e.* decreasing diffraction intensity starting with high-resolution reflections and increasing Wilson and scaling *B* factors, *R* factors, mosaicity and unit-cell volume. Global effects can perhaps be explained by the production of hydrogen gas (Meents *et al.*, 2009 ▶, 2010 ▶), but the physico-chemical nature of radiation damage remains unexplained.

Carpentier *et al.* (2010 ▶) combined Raman spectroscopy with X-ray studies of chicken egg-white lysozyme (CEWL) crystals to study damage to the disulfide bond. They proposed a process initiated by a rapid build-up of an anionic radical intermediate that either reverts back to the oxidized state or evolves towards a protonated radical species or a cleaved product. Their data strongly suggested an X-ray-induced ‘repair’ mechanism. This was supported by previous UV–visible microspectrometry studies on the X-ray irradiation of trypsin crystals (McGeehan *et al.*, 2009 ▶).

In the work presented here, radiation chemistry theory is combined with X-ray diffraction measurements, UV–visible absorption spectroscopy and electron paramagnetic resonance (EPR) to develop and test a quantitative model of the radiation damage associated with disulfide bonds. While CEWL crystals are used to test the model, the model itself is based on physical-chemical properties and therefore its application is not limited to lysozyme. It is proposed as a first step in a comprehensive predictive model of the radiation-induced processes that perturb the native structure of macromolecules.

## 2. The radiation chemistry of disulfide-bond breakage   

### 2.1. Mechanistic model   

In earlier work on crystals and films of DNA, a mechanistic model was developed to describe the dose-dependence of radiation products. The model was used to quantitatively connect the yields of product (produced by 70 keV X-­rays) with the yields of the intermediate free radicals trapped by DNA (Swarts *et al.*, 2007 ▶). These products were produced as a result of energy being deposited directly in the DNA; doses of 10–100 kGy were required to detect and quantify the products. In this dose range, a transition in the dose-response curve of the product was difficult to explain using a conventional model based on a one-to-one correspondence between radical intermediate and end product. A new model was developed that ascribes product formation in the higher dose range to the interaction of two separate events (Swarts *et al.*, 2007 ▶). At incident X-ray energies typically associated with macromolecular crystallography, up to a recommended maximum absorbed dose of 30 MGy (Owen *et al.*, 2006 ▶), the photoelectric effect dominates. This creates a fast electron along with an associated cation. The photoelectron propagates along a track creating additional energetic electrons and cations. The ejected electrons eventually thermalize, primarily creating anions. The resulting track is a branched inhomogeneous distribution of anions, cations and excitations. As the dose increases, the probability of one track overlapping with another increases and consequently the probability of any given site being ionized twice also increases. The two separate events that are featured in the new model come from the interaction of multiple tracks and the model is therefore termed the multi-track model.

Under the expectation that the two ionizations at one site would play an important role in explaining the disulfide-bond (S—S) damage observed in X-ray crystallographic studies of macromolecular crystals, here the original model is expanded and developed to describe these systems. The hypothesis is that the S—S bond in macromolecular crystals at 100 K would not be cleaved as a result of a single one-electron reduction but rather by one-electron reduction followed by protonation then a second one-electron reduction. Concurrence of these events at the same site, although possible within a single track of ionizing radiation, has a much higher probability when tracks overlap one another. The probability of S—S bond cleavage therefore increases when two tracks intersect at the same S—S site.

**Figure 1:**
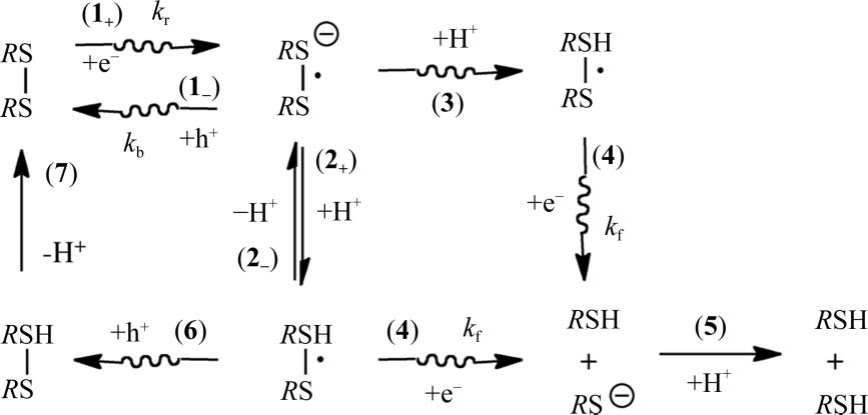
The reaction pathways constituting the proposed chemical model for cleavage of the disulfide bond.

The key reaction pathways constituting our chemical model for cleavage of the disulfide bond (*R*SS*R*, where *R* is used to denote the remainder of the cysteine residue) into sulfhydryl groups (*R*SH + *R*SH) in the solid state (with crystals at ≤130 K) are shown in Fig. 1 ▶. In step **1_+_**, one-electron addition yields the radical anion 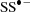 (also termed 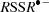) with the rate constant *k*_r_. If *R*SS*R* is coordinated with a favorable proton donor, then proton transfer gives the neutral radical 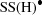, as in step **2_+_**. This is reversible, with the back-reaction indicated in Fig. 1 ▶ as step **2_−_** providing a repair pathway. If a radical cation is generated in the proximity of *R*SS*R*, either by the same track or a second track, deprotonation of this radical cation may result in the protonation of 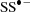 [to become 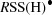]: this is presented as step **3** in Fig. 1 ▶. Unlike step **2_+_**, step **3** is not reversible. The unpaired electron in 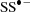 and 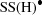 resides in a three-electron σ bond (Rao *et al.*, 1983 ▶; Asmus *et al.*, 1977 ▶).

In our system, the 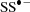 and 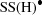 radicals will be highly reactive with holes (radical cations designated h+) and electrons that are generated by an overlapping track. Reaction with a hole generated by the same or a second track takes the radical anion backwards to its parent (step **1_−_** in the case of a deprotonated radical or step **6** for the protonated radical species). On the other hand, electron attachment (step **4**) drives 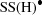 forward to the product with a rate constant *k*_f_. The cleavage products, *R*SH and *R*S^−^, can progress *via* step **5** to give *R*SH + *R*SH. Pivotal to S—S cleavage is the competition between the back-reaction at rate *k*_b_ in step **1_−_** and the forward reaction at rate *k*_f_ in step **4**.

### 2.2. Mathematical description   

Our mathematical model, based on the reaction scheme proposed in Fig. 1 ▶, consists of two first-order reactions. In the first, radiation drives a reversible reaction between a parent molecule *M* and a radical intermediate *R*, and in the second, radiation irreversibly drives the radical *R* to the product *P*. The first step occurs through reaction **1_+_** and is further stabilized through the protonation reaction **2_+_** (Fig. 1 ▶). The second step occurs through reaction **4** and is stabilized by protonation reaction **5**,
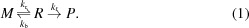



In this case *M* is the disulfide bond, *R*SS*R*, and *P* is the cleaved disulfide, which results in two *R*SHs. The identity of the radical intermediate *R* depends on the path. All of the reactions proposed in Fig. 1 ▶ fall into the categories of either radicalization or product formation. The dependence of the concentration of *M*, *R* and *P* on dose, *D*, is described by three first-order differential equations containing rate constants *k*_r_, *k*_f_ and *k*_b_,
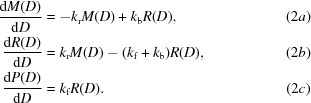



The solutions are
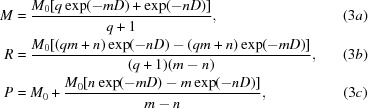
where *M*_0_ is the concentration of *M* at zero dose and *m*, *n* and *q* (used for compactness) are determined from the three rate constants as
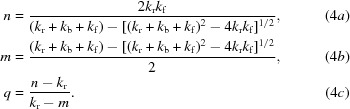



(2)–(4) describe the dose dependence of the concentrations of *M*, *R* and *P* using four physically relevant parameters: the rate constants *k*_r_, *k*_f_ and *k*_b_ and the initial concentration of *M*, *M*_0_. It is important to note that (3*a*), (3*b*) and (3*c*) satisfy the physical properties of the system. For instance, at zero dose *M* = *M*_0_, *R* = 0 and *P* = 0. When *D* is extremely large, *M* approaches 0, as does *R*, while *P* approaches *M*_0_, accurately representing the total depletion of *M* and *R* at high dose as it is converted to final product *P*.

## 3. Experimental testing of the model   

### 3.1. Crystal preparation   

CEWL crystals for the electron paramagnetic resonance (EPR) and X-ray crystallo­graphic experiments were prepared using protein purchased from Hampton Research without further purification. Crystals were grown using the hanging-drop vapor-diffusion method with a protein concentration ranging from 50 to 75 mg ml^−1^ in 0.1 *M* sodium acetate buffer pH 4.8. The precipitant, also in the same buffer, contained 7.5–15% sodium chloride and 25% ethylene glycol as a cryoprotectant agent. Drops of 10 µl were set up with a 1:1 protein:precipitant ratio. For the UV–visible spectroscopy (microspectrophotometry) studies, similar crystallization conditions were used with the exception that the cryoprotectant agent was incorporated by soaking crystals in mother liquor containing 20%(*v*/*v*) glycerol (water replaced by glycerol) rather than growing the crystals in the presence of ethylene glycol.

### 3.2. UV–visible microspectrophotometry   

Eight crystals were mounted in separate nylon loops (Hampton Research, Aliso Viejo, California, USA), flash-cooled and held at 100 K by an open-flow nitrogen stream, and were then irradiated on beamline I24 at Diamond Light Source. X-rays of energy 12.8 keV were used and the beam was defocused to 50 × 50 µm with incident fluxes ranging from 8.58 × 10^9^ to 1.54 × 10^12^ photons s^−1^ at the sample position (filter transmission from 0.8 to 100%). This resulted in dose rates ranging from 1.5 to 2700 kGy s^−1^. Each crystal was subjected to a single X-ray exposure, the duration of which varied such that the total absorbed dose was ∼5 MGy. All doses were calculated using *RADDOSE* v.2 (Murray *et al.*, 2004 ▶; Paithankar *et al.*, 2009 ▶). An initial crystal orientation was chosen to yield the cleanest spectroscopic signal and changes in UV–visible optical absorbance were measured using an *in situ* microspectrophotometer with a 50 µm diameter probe beam to closely match the X-ray illuminated area. Spectra were collected using mirrored lenses (Bruker) mounted in an off-axis geometry with a deuterium halogen light as the light source (Ocean Optics). Absorption was monitored over the 300–800 nm wavelength range using a Shamrock 303 imaging spectrograph (Andor). Spectra were recorded every 200 ms for the duration of the experiment and for a period after the shutter was closed. To further probe saturation effects, an additional crystal was subjected to a series of 1 s exposures interspersed with 5 s rest periods during which the X-ray shutter was closed.

### 3.3. Irradiations and EPR   

For the EPR studies, the crystals were harvested directly from the crystallization drop and mounted in 1.0 mm outer diameter thin-walled quartz glass capillaries (Hampton Research, Aliso Viejo, California, USA). An approximate measure of crystal dimensions was made for each (±50 µm) using a stereo microscope. The capillaries were sealed at either end using wax and mounted on a sample stage for the EPR measurements. These were inserted one at a time into a Janis liquid-helium cryostat in the EPR instrument and cooled to a temperature of 4 K in less than 30 s (the cryostat maintains temperature through expansion of liquid helium into a vacuum environment). No attempt was made to obtain precise information on the alignment of the crystals with respect to the magnetic field. Crystals were irradiated *in situ* with median energy 50 keV X-rays at 4 K using a Varian/Eimac OEG-76H tungsten target tube operated at 70 kV, 20 mA and filtered by a 25 µm aluminium foil. The dose rate at the sample was 0.0125 kGy s^−1^ as determined by calibration with radiochromic film (Niroomand-Rad *et al.*, 1998 ▶). Following irradiation, EPR data collection was performed on samples at 4 K. First-derivative EPR absorption spectra were recorded at the Q-band (35.3 GHz) microwave frequency. An upper limit on the formation of water ice during sample cooling was obtained by the observation that the EPR signal owing to H atoms was not observed. Ice irradiated at 4 K gives a distinctive 50 mT doublet owing to trapped H atoms (Johnson & Moulton, 1978 ▶). Lack of the doublet signal implies a limit on the ice content of a few percent of the crystal mass and, based on extensive experience with other organic samples at high concentration, the cooling procedure used created little to no water ice. As the system is closed, we cannot absolutely determine whether the sample has cooled amorphously or whether crystalline ice has formed. However, the EPR signal is largely independent of this (Bednarek *et al.*, 1998 ▶), so unlike during crystallographic studies, the type of ice formed does not impact on the measurements.

Double integration of the EPR spectra gave the number of trapped free radicals by comparison with the signal from a ruby standard mounted on the inside wall of the microwave cavity. A relatively weak quartz signal produced by radiation in the capillary was subtracted out as described previously (Purkayastha & Bernhard, 2004 ▶) and thereby the signal owing to the lysozyme crystal alone was isolated. The number of radicals per crystal mass is the radical concentration *R*(*D*) used to calculate the chemical yield. A total of three lysozyme crystals were studied; their dimensions, approximate volumes and the dose points at which spectra were recorded are given in Table 1 ▶. The mass of crystal 1 was determined by weighing the crystal sealed in the capillary before irradiation, repeating the measurement after irradiation with the capillary cracked to remove any mother liquor and finally dissolving the crystal and weighing just the dry capillary and wax sealant. The masses of the other two crystals were determined by scaling the known mass of the first crystal to the total free-radical concentration recorded at a dose of 20 kGy. These masses were validated by comparison with the measured dimensions (Table 1 ▶).

After accumulating data at 4 K, crystal 3 was annealed to consecutively higher temperatures (50, 100 and 150 K), held at that temperature for 15 min and then returned to 4 K (where all radicals are trapped) to record the impact of annealing.

Chemical yield is defined as the slope of the dose response at zero dose. The dose response for radicals trapped in the solid can be described by
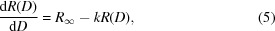
the solution of which is
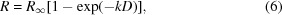
where *R* is the radical concentration, *k* is the dose-dependent rate of radical destruction and *R*_∞_ is the radical concentration at infinite dose (Nelson, 2005 ▶; Snipes & Horan, 1967 ▶). In order to obtain the expression for the chemical yield *G*, a Taylor expansion is applied under the condition *kD* << 1 (valid when destruction of the radical only returns it to the parent structure, *i.e.* zero to low doses), such that
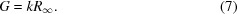



Under these conditions *R* reaches saturation at *R*_∞_. The units of *R* are mol g^−1^ and the initial slope of the dose response, *R* *versus* *D*, is the chemical yield *G* in mol J^−1^.

Of interest here are the two closely related radicals 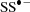 and 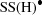 described in §[Sec sec2.1]2.1 and shown in Fig. 1 ▶. 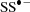 and 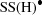 are collectively described by the radical concentration denoted *R*(SS). The fraction of trapped radicals ascribed to *R*(SS) is *F*(SS) = *R*(SS)/*R*(tot), where *R*(tot) is the concentration of all radicals trapped in the crystal. The free-radical yields associated with *R*(SS) and *R*(tot) are *G*(SS) and *G*(tot), respectively, each with units of mol J^−1^.

The dose-response curves were recorded for the three separate crystals described earlier. The yield of all radicals trapped in each crystal, *G*(tot), was calculated from the initial slope of the curve described by (6). Nonlinear least-squares fits were performed using *GraphPad Prism* (GraphPad Software, San Diego, California, USA).

In order to determine *F*(SS), the powder spectrum of 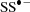 was simulated using published *g*− and hyperfine coupling tensors reported by Lawrence *et al.* (1999 ▶), and the simulated spectrum was used to fit the *R*(SS) component of the EPR spectrum by matching the high-*g* component of the disulfide radical signal to the high-*g* spectral component of the simulated signal. The use of a powder spectrum assumes a large number of randomly oriented radicals. Simulations were performed using *Powder Sim*, a program developed in-house (Bernhard & Fouse, 1989 ▶). While treating the experimental spectrum as a powder spectrum is not strictly correct, it is a reasonable approximation because (i) the lysozyme crystal contains 32 magnetically distinct cysteines per unit cell (four per molecule and eight molecules per unit cell) and (ii) the signal anisotropy was difficult to discern when the crystals were rotated through 180° in 15° steps. Of course, (ii) is a direct consequence of (i).

### 3.4. X-ray crystallography   

For crystallographic studies, crystals were harvested using nylon loops (Hampton Research, Aliso Viejo, California, USA) and flash-cooled in liquid nitrogen. They were shipped to the Stanford Synchrotron Radiation Laboratory (SSRL; Palo Alto, California, USA), where diffraction data were collected at 100 K remotely using a MAR325 CCD detector on beamline 9-2. The data were collected at an X-ray energy of 12 keV (1.033 Å) and a crystal-to-detector distance of 131.6 mm, and the beam was attenuated by 93.6%, giving a flux of 3.8 × 10^10^ photons s^−1^. Two initial images were recorded 90° apart and were used with the *STRATEGY* option of *Blu-­Ice* (González *et al.*, 2008 ▶; McPhillips *et al.*, 2002 ▶) to define an appropriate starting angle. A total of 15 data sets over 57° were collected using a 2 s exposure and an oscillation angle of 1° per image, with each data set starting at the same position as the first, thus ensuring that the same area of the crystal was irradiated during each data set. The crystal was approximately 0.3 × 0.3 × 0.3 mm in size, with the beam (approximating a top-hat profile) illuminating an area of 0.2 × 0.2 mm. The absorbed dose was estimated using the program *RADDOSE* v.2 (Murray *et al.*, 2004 ▶; Paithankar *et al.*, 2009 ▶) but was not adjusted for fresh regions of the crystal that rotated into the beam (estimated to reduce the calculated absorbed dose by less than 0.2% per degree).

The data were integrated with *HKL*-2000 (Otwinowski & Minor, 1997 ▶) and reduced with *SCALA* (Evans, 2006 ▶). An initial model was obtained by molecular replacement using *MOLREP* (Vagin & Teplyakov, 2010 ▶) with the structure of lysozyme (PDB entry 6lyz; Diamond, 1974 ▶) as the search target. The resulting model was refined against the data using an iterative process combining *PHENIX* (Adams *et al.*, 2010 ▶) with manual model building using *Coot* (Emsley *et al.*, 2010 ▶). The process continued until there were no unexplained positive or negative peaks in the electron density above 5σ. Isomorphous difference Fourier maps, *F*_o,*n*_ − *F*_o,1_ (Rould & Carter, 2003 ▶) were calculated with *PHENIX* (Adams *et al.*, 2010 ▶) using the observed amplitudes from each data set and the phases derived from the model fitted to the first data set. This technique is a sensitive way to visualize specific damage (Weik *et al.*, 2000 ▶; Carpentier *et al.*, 2010 ▶). The maps were viewed in *CCP*4*mg* (McNicholas *et al.*, 2011 ▶). The solvent accessibility of the cysteine residues involved in the disulfide bonds was calculated using *AREAIMOL*, part of the *CCP*4 package (Winn *et al.*, 2011 ▶).

## 4. Results   

### 4.1. UV–visible microspectrophotometry   

**Figure 2:**
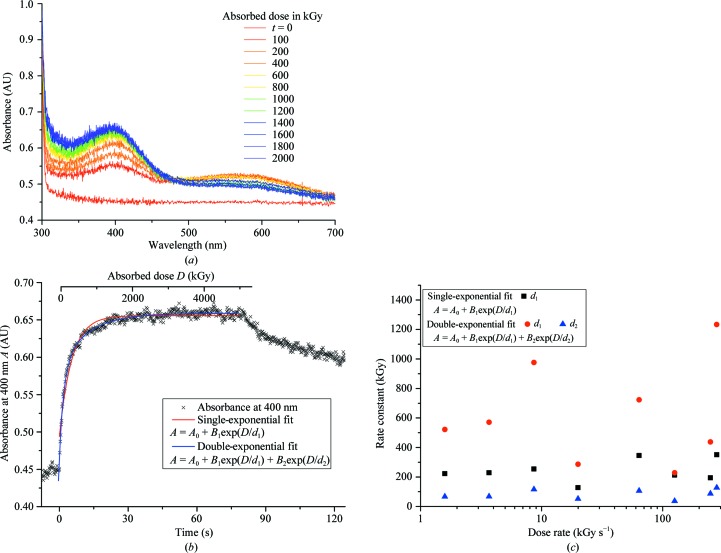
The dose-response behavior of the 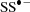 radical in a lysozyme crystal observed through UV–Vis absorption spectroscopy. (*a*) The spectra show the rapid rise in the overall signal owing to the increase in radical concentration and the temporal evolution of absorption peaks at 400 and 580 nm. An isosbestic point is present at 480 nm. (*b*) Absorbance at 400 nm (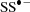 radical signal) as a function of absorbed dose with single- and double-exponential fits overlaid (residuals are shown in the Supplementary Material). The crystal was subjected to a total absorbed dose of ∼5 MGy (∼80 s at 61.8 kGy s^−1^) before the shutter was closed (see text for details). (*c*) Variation in fit parameters as a function of dose rate; no systematic trend is observed.

X-ray-induced changes in the optical absorption of lysozyme crystals upon irradiation were monitored using an online microspectrophotometer as described above. The increased absorbance at 400 nm is attributable to the radical species 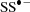 (Weik *et al.*, 2002 ▶; Southworth-Davies & Garman, 2007 ▶) and an increase in absorbance at this wavelength was clearly observed in all samples. This was accompanied by a peak in absorption at ∼580 nm (Fig. 2 ▶ *a*) which is attributable to the formation of solvated electrons (McGeehan *et al.*, 2009 ▶). Both of these features can clearly be seen in the spectral series in Fig. 2 ▶(*a*), which shows the results of a continuous 80 s irradiation with a cumulative dose of 5 MGy (dose rate of 62 kGy s^−1^). The absorbance at 400 nm increases rapidly before saturating and the 580 nm peak owing to solvated electrons has an observed maximum at the earliest recorded point. This peak may have been higher at earlier time points (below 200 ms) that were not captured in the experiment. The observation that this solvated electron signal (580 nm) decreases as the 400 nm absorption peak increases supports our model; the solvated electrons are depleted as 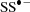 and other one-electron reduction products are formed. This is in agreement with a related study on lysozyme by Allan *et al.* (2013 ▶) also using UV–visible microspectrophotometry. Allen and coworkers observed an initial rise in the 580 nm absorption with increasing dose, followed by a fall in this signal corresponding to an increase in absorption at 400 nm. In Fig. 2 ▶(*b*)^1^ the dose-dependent increase in absorbance at 400 nm is plotted. The dose-response curves were fitted to both a single- and a double-exponential function Abs = *A*_0_ + *B*_1_exp(*D*/*d*_1_) + *B*_2_exp(*D*/*d*2), where *A*_0_ is the baseline, *B*_1_, *B*_2_, *d*_1_ and *d*_2_ are constants and *D* is the dose. For the double-exponential fit *d*_1_ and *d*_2_ were defined such that *d*_1_ > *d*_2_. *B*_2_ was defined as zero for the single-exponential fit. All data could be well fitted with a single or double exponential with an *R* ^2^ of ≥0.95, although visual inspection of the fits showed that the double-exponential parameterization better describes the data (Fig. 2 ▶ *b*). The constants *d*_1_ and *d*_2_ are shown as a function of dose rate in Fig. 2 ▶(*c*) for both single- and double-exponential fits. We define the saturating dose, *D*_90_, as the point at which the absorbance reaches 90% of the maximum above baseline. This is the dose at which fast changes no longer dominate. In this case the *D*_90_ for lysozyme crystals averages 0.51–0.77 MGy (depending on the single- or double-exponential fit), but the variability is large (see Table 2 ▶). There was no clear indication of dose-rate dependence on the saturation level.

**Figure 3:**
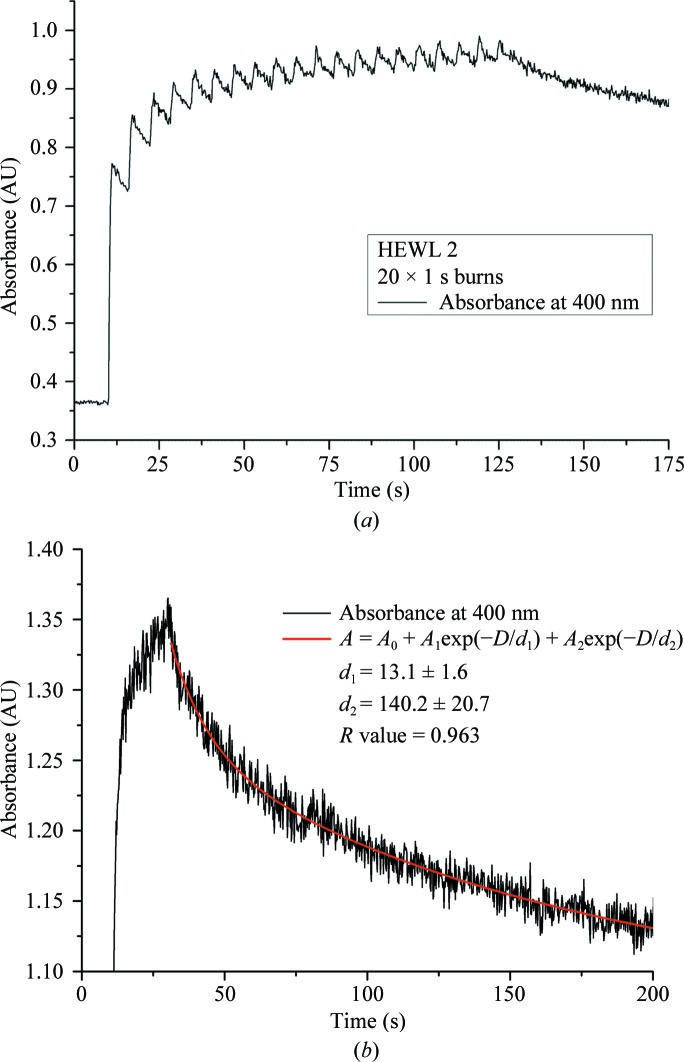
Absorbance measured for (*a*) 20 × 1 s burns and (*b*) a single 20 s burn. The absorbed dose per 1 s exposure was 287 kGy. The cumulative dose over 20 s was thus 5.74 MGy. (*a*) The multiple burns show a progressively smaller change in absorption for the same absorbed dose and rapid loss of 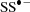 was observed after each pulse. (*b*) The single continuous 20 s burn highlights the post-exposure decay of the disulfide peak, which is best described by a two-rate model, in agreement with previous observations (Owen *et al.*, 2011 ▶; Beitlich *et al.*, 2007 ▶).

The change in absorbance from a series of 1 s exposures interspersed with a 5 s rest period is shown in Fig. 3 ▶(*a*). Despite a rapid reduction in absorbance when the X-ray shutter was closed for the rest period, saturation at 400 nm was still achieved swiftly with a progressively smaller change in absorption for the same additional absorbed dose. The reduction in absorption seen during the rest period indicates that some fraction of 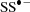 was lost owing to recombination and/or deprotonation, but the dominating increase over time indicates that some fraction was stable at 100 K. The post-exposure decay of the disulfide peak at 400 nm subsequent to a 20 s continuous X-ray exposure is shown in Fig. 3 ▶(*b*). The decay follows a double-exponential form with rate constants *d*_1_ and *d*_2_ equal to 13.1 ± 1.6 and 140.2 ± 20.7 s^−1^, respectively (Fig. 3 ▶ *b*). The fit of the decay by a double-exponential function is in agreement with previous observations (Owen *et al.*, 2011 ▶; Beitlich *et al.*, 2007 ▶). Both results, Figs. 3 ▶(*a*) and 3 ▶(*b*), add support to the multi-track model comprising both product formation and destruction.

### 4.2. Irradiations and EPR   

Radical trapping in lysozyme crystals at 4 K was quantified using EPR spectroscopy for three different lysozyme crystals, as detailed in Table 1 ▶. Crystal 1 sampled absorbed doses from 5 to 150 kGy, with crystal 2 used to replicate similar doses. Crystal 3 extended the absorbed dose range to a total dose of 500 kGy. Crystal 1 weighed 208 µg and the calculated weights of crystals 2 and 3 from the total free-radical concentration at 20 kGy were 135 and 185 µg, respectively. These were compatible with the observed crystal volumes and allowed normalization of the data, enhancing the analysis based on relative changes as a function of dose. However, it should be noted that the absolute free-radical yield is based on the data set from crystal 1 only. The yield of one-electron reduced *R*SS*R*, denoted *G*(SS), was calculated using the *R*(SS) component of the spectrum.

**Figure 4:**
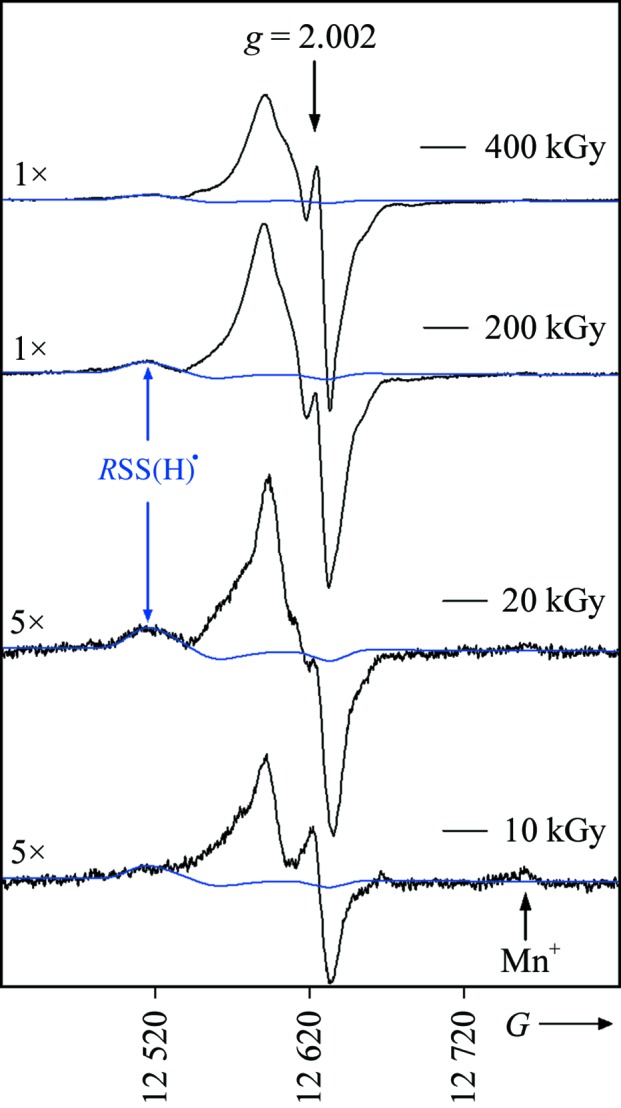
Four Q-band EPR spectra (in black) recorded for crystal 3 at 4 K after X-­irradiation at 4 K. The first two dose points of 10 and 20 kGy have been scaled by ×5 for clarity. The scan width is 40 mT. The orientation of the crystal was not determined. The simulated spectrum of 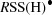 is shown in blue with the high-*g* peak explicitly labeled for 20 and 200 kGy dose points. The sharp peak at *g* ≃ 2.002 becomes apparent at doses above 100 kGy; this is well characterized and is owing to paramagnetic centers trapped in the quartz sample holder. At high field a weak signal is observed in the 10 and 20 kGy spectra (marked by an arrow). This signal is owing to trace amounts of Mn^+^.

**Figure 5:**
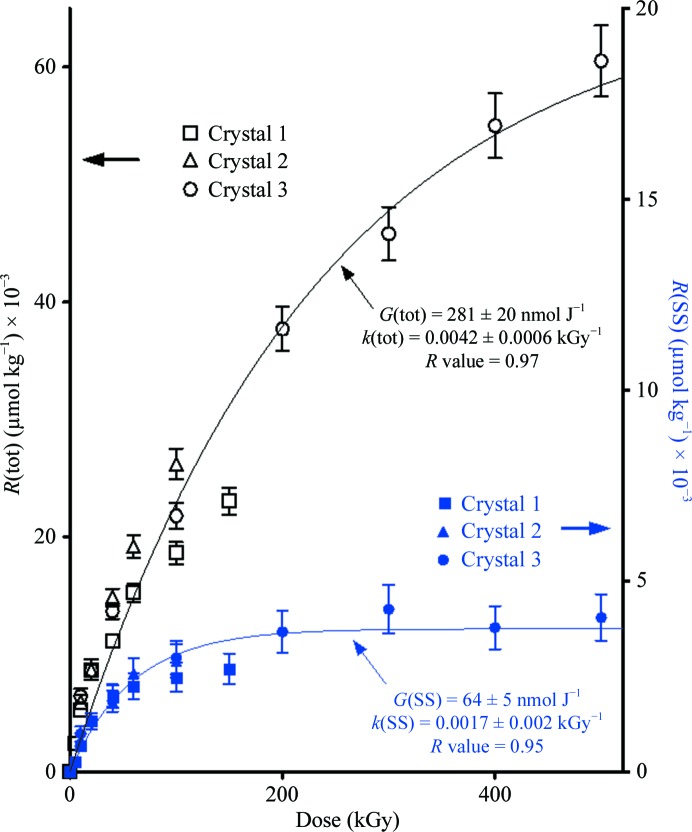
The dose response for radical trapping in lysozyme crystals irradiated with 70 keV X-rays at 4 K. Data for the concentration of total trapped radicals, *R*(tot), are shown for three different crystals using open black symbols (left *y* axis). Data for radicals formed by reduction of *R*SS*R*, *R*(SS), are shown using closed blue symbols (right *y* axis). See text regarding the normalization of the data using the measured mass of crystal 1. The curves were obtained by a nonlinear least-squares fit of the data using equations (6) and (7) with the parameters detailed in the figure.

In Fig. 4 ▶, EPR spectra are shown for four different X-ray doses. At low doses in the EPR experiment, *e.g.* between 10 and 20 kGy, the spectrum intensity increases linearly with dose. At higher doses, *e.g.* 200–400 kGy, a plateau is reached. The blue traces in Fig. 4 ▶ are simulations of the 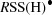 component, which as described above is associated with the low-field signal assigned exclusively to 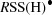. The double integral of the experimental and calculated spectra gave the radical concentrations *R*(tot) and *R*(SS), respectively. These concentrations were used in the dose-response curves shown in Fig. 5 ▶. In Fig. 4 ▶ the peak from the growing 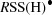 component is indicated along with a peak from trace amounts of Mn^+^ known to be present in the experimental setup.

In Fig. 5 ▶, the *R*(tot) data are plotted using black symbols referring to the left *y* axis and the *R*(SS) data are plotted using blue symbols referring to the right *y* axis. The curves fitting these data are derived from a nonlinear least-squares fit to (6). The fitting parameters for *R*(tot) were *G*(tot) = 281 ± 20 nmol J^−1^ and *k* = 4.2 ± 0. 6 MGy^−1^. For *R*(SS), the fitting parameters were calculated to be *G*(SS) = 64 ± 5 nmol J^−1^ and *k* = 17 ± 2 MGy^−1^. The saturation values for *R*(SS) *versus* *R*(tot) are distinctly different, reflecting the differences in dose-response properties between the radical species. *R*(SS) saturates at ∼200 kGy at a value of *R*(SS)_∞_ = 3.7 ± 0.5 mmol kg^−1^, whereas *R*(tot) saturates above 500 kGy at a value of *R*(tot)_∞_ = 66 ± 10 mmol kg^−1^. This difference is a consequence of the relatively large destruction cross-section for the SS-centered radicals compared with those of the other radical species trapped in lysozyme.

The above values for *G*(SS), *k* and *R*(SS)_∞_ are for the sum of all four disulfide bonds in lysozyme. EPR data cannot distinguish between the individual SS sites; however, division by 4 [*G*(SS)/4 = 16 nmol J^−1^] provides an average yield at each site, with the crystallographic data being used to explore differences between sites in detail.

In terms of (1), *M* is the concentration of *R*SS*R* and is denoted *M*(SS). Using a density of 1.17 g cm^−3^, the concentration of cystine, [*R*SS*R*], based on the lysozyme crystal structure was calculated to be 229 mmol kg^−1^. *R* is *R*(SS), the concentration of SS radicals, and *P* is the concentration of product resulting from cleavage of the S—S bond, which is denoted *P*(SS*). Since we do not have a direct measure of *P*(SS*), *M*_0_ − *M* is used as a measure of SS*. It is assumed that the decrease in occupancy by one of the two sulfurs in *R*SS*R* is equal to *P*(SS*). The S atom chosen is that whose occupancy is most sensitive to dose, the logic being that loss of occupancy of either of the S atoms forming the S—S bond implies that the bond was broken. With respect to product formation, the rate-limiting step in the reaction scheme shown in Fig. 1 ▶ is postulated to be a one-electron reduction of 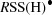. Consequently, whether proton transfer is thermodynamically (**2_+_**) or radiation (**3**) driven, product formation is governed solely by *k*_f_ and *k*_b_. Another rate constant to account for reaction **3** could be included. However, the experimental data suggest that the one-electron reduction rate-limiting step is correct and that the reaction kinetics are dominated by processes **1_−_** and **4** (Fig. 1 ▶). A third rate constant would have only marginal effects.

During the EPR annealing experiments, no spectral changes occurred until a temperature of 130 K was reached. This observation indicates that processes observed at 4 K by EPR can be directly related to experimental X-ray crystallographic data-collection conditions at 100 K. At 130 K changes in the EPR spectral features were observed, but these changes were indicative of thermal evolution of radical species distinct from the disulfide radical anion. The signal from the disulfide radical anion persisted up to a temperature of 190 K.

### 4.3. X-ray diffraction data and structural results   

The statistics for the diffraction data collection at 100 K and the structural refinement results are summarized in Table 3 ▶. 15 consecutive data sets were collected from a single tetragonal *P*4_3_2_1_2 crystal (similar to that used for the microspectro­photometry studies) and the dose per data set was 0.07 MGy, with a cumulative dose of 1.05 MGy. Beyond a progressive increase in scaling *B* factors from 10.7 to 11.3 Å^2^ there were no systematic trends in the crystallographic statistics as a function of absorbed X-ray dose, and few global indicators of damage were observed. The unit-cell parameters remained approximately constant and the signal-to-noise [*I*/σ(*I*)] in the highest resolution shell decreased slightly from 5.0 to 4.7. In terms of structural refinement statistics for each model (*R*_work_ and *R*_free_) there were also no global differences between models independently derived from the different data sets collected as a function of dose. Structurally, apart from a slight increase in the S—S bond distance, there were no major differences between data sets.

**Figure 6:**
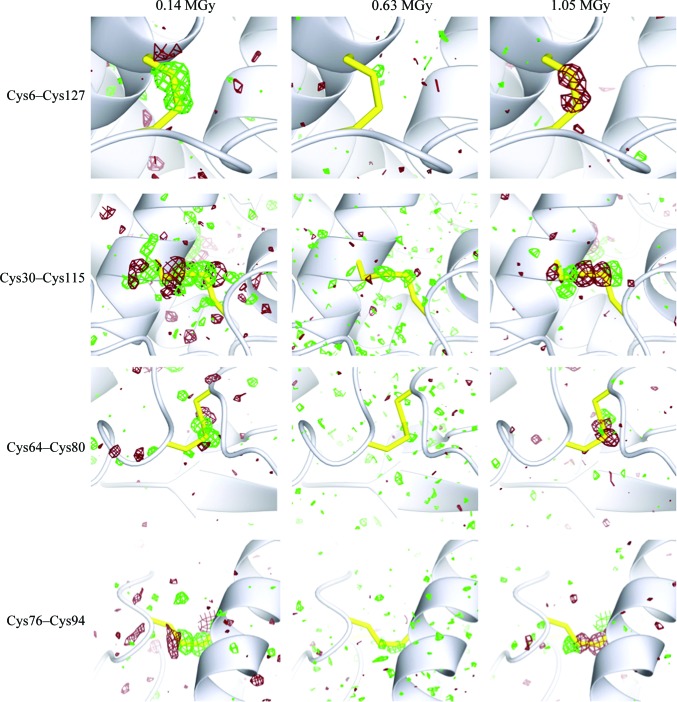
Isomorphous difference density maps *F*_o,*n*_ − *F*_o,1_ (where *n* is the data-set number) around the four disulfide bonds present in lysozyme. Maps are shown for *F*_o,2_ − *F*_o,1_ (0.14 MGy), *F*_o,9_ − *F*_o,1_ (0.63 MGy) and *F*_o,15_ − *F*_o,1_ (1.05 MGy). Disulfide bonds are highlighted in yellow. Maps are contoured at +3σ (green) and −3σ (red). For Cys6–Cys127 the topmost part of the bond is Cys6, with the bottom being Cys127. The remaining bonds are positioned such that the label matches the residue positions in each figure, with the first to the left and the second to the right. Note that the dose indicated is the cumulative dose.

*F*_o,*n*_ − *F*_o,1_ maps contoured at 3σ for the disulfide bonds at each successive whole data-set dose are shown in Fig. 6 ▶. Positive density (green) indicates the presence of more electron density than seen in the 0.07 MGy data set, while negative density (dark red) results when the opposite is true. An inspection of the successive-dose electron-density maps in the area associated with each disulfide bond showed bond-specific effects.

CEWL is a relatively small protein with 129 residues and four disulfide bonds per molecule. Two of these are intra-α-­domain disulfide bonds (Cys6–Cys127 and Cys30–Cys115), one is an intra-β-domain bond (Cys64–Cys80) and the final one is an inter-αβ-domain bond (Cys76–Cys94). The two intra-α-domain disulfides, Cys6–Cys127 and Cys30–Cys115, appeared to be more sensitive to radicalization, with positive density immediately visible in the *F*_o,2_ − *F*_o,1_ map at an absorbed dose of 0.14 MGy. As dose increases this electron density dissipates, with negative density (presumably the initial signs of bond breakage) appearing in the *F*_o,12_ − *F*_o,1_ map (0.84 MGy; not shown) and clearly observed in the *F*_o,15_ − *F*_o,1_ map at 1.05 MGy. For the Cys30–Cys115 bond there is initial evidence of both positive and negative density in the region of the bond. The positive density directly surrounds the bond, while the negative density seems to be at either end of the two S atoms making it up. The positive density is again indicative of a radicalization, while the negative density suggests that a positional shift is occurring. As the dose increases, the effect is reversed; negative density indicates bond breakage, clearly seen at 1.05 MGy, while positive density suggests the new positions of the sulfurs.

The intra-β-domain disulfide Cys64–Cys80 again shows positive electron density in the *F*_o,2_ − *F*_o,1_ map, with negative density (possibly the start of bond breakage) appearing at 0.77 MGy and indicated in the 1.05 MGy *F*_o,15_ − *F*_o,1_ map. The inter-αβ-domain Cys76–Cys94 disulfide also shows negative density immediately but the progression as a function of dose is small; Cys76–Cys94 seems to be the least susceptible bond to cleavage. This bond is stabilized by a weak hydrogen bond, providing a proton source, between the S atom of Cys94 and a water molecule. While this appears to be least susceptible to cleavage, weak electron density consistent with an alternate conformation of the rotamer of Cys94 appears to develop with increasing dose.

Overall, the Cys64–Cys80 and Cys76–Cys94 bonds appear to be less affected than Cys6–Cys127 and Cys30–Cys115. The solvent surrounding the protein molecule is a potential source of free radicals and the accessibility of the residues associated with each bond has been calculated and is detailed in Table 4 ▶. The residues in the Cys6–Cys127 bond are the most solvent-accessible of the four disulfide bonds, while Cys30–Cys115 has a small accessible area for both Cys30 and Cys115, and Cys64–Cys80 has a larger area for Cys64 but no solvent-accessible area for Cys80. Solvent-accessible area does not appear to have a direct connection with the damage observed, as previously noted by Fioravanti *et al.* (2007 ▶) in a study of radiation-induced decarboxylation of glutamates and aspartates.

The *F*_o,*n*_ − *F*_o,1_ maps are sensitive to small changes. However, it should be remembered that these maps are based on the difference from the model produced from the first set of diffraction data. If the bond was already becoming radicalized at 0.07 MGy, then the positive electron density seen is an underestimate since the initial signs of bond breakage would occur earlier. From the crystallo­graphic data alone we cannot determine whether this is the case. Sensitivity to radiation damage in the Cys6–Cys127 region has been noted in other studies at both cryogenic temperatures (Weik *et al.*, 2000 ▶) and ambient temperature (Kmetko *et al.*, 2011 ▶).

**Figure 7:**
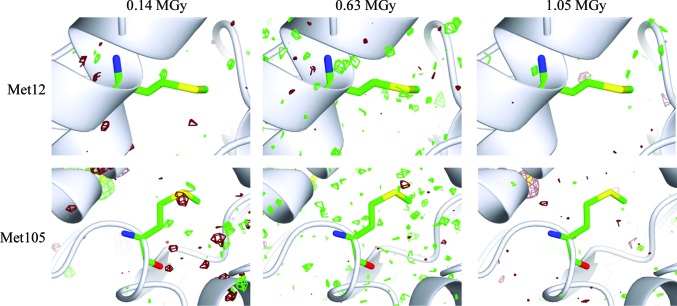
Isomorphous difference density maps *F*_o,2_ − *F*_o,1_ (0.14 MGy), *F*_o,9_ − *F*_o,1_ (0.63 MGy) and *F*_o,15_ − *F*_o,1_ (1.05 MGy) for residues Met12 and Met105. Maps are contoured at 3σ in green and −3σ in dark red.

In addition to the S atoms in the four disulfide bonds, there are also two additional S atoms present in lysozyme in Met12 and Met105. Both of these have zero solvent accessibility. The *F*_o,*n*_ − *F*_o,1_ maps for these residues are shown in Fig. 7 ▶. For Met12 there is little if any indication of dose-related damage and negligible initial damage of the carbon–sulfur bond in Met105. This possible localized damage on the S atom is present in the *F*_o,*n*_ − *F*_o,1_ maps from the initial map to *F*_o,6_ − *F*_o,1_ (0.49 MGy). However, after the sixth data set it is no longer localized. Overall, the effect appears to be marginal.

The resulting structures and experimental data have been deposited in the PDB as entries 4h8x, 4h8y, 4h8z, 4h90, 4h91, 4h92, 4h93, 4h94, 4h9a, 4h9b, 4h9c, 4h9e, 4h9f, 4h9h and 4h9i, with the absorbed doses starting at 0.07 MGy for 4h8x and incrementing by 0.07 MGy to 1.05 MGy for 4h9i.

## 5. Discussion   

In this study, for the first time, online microspectro­photo­metry, EPR with *in situ* X-ray irradiation and X-ray crystallography have all been combined to study disulfide damage in lysozyme crystals. This has allowed us to sensitively probe the radical chemistry at low doses with EPR, while UV–visible microspectrophotometry permitted the investigation to be extended to conditions typical for cryocrystallographic studies. Finally, the crystallographic studies allowed us to probe site-specific effects that are averaged in the EPR and spectroscopic analyses.

UV–visible spectroscopy showed that disulfide radicalization appeared to saturate at an absorbed dose of approximately ∼0.5–0.7 MGy (depending on the fit), in contrast to the saturating dose of ∼0.2 MGy observed by EPR at a much lower (in the largest case by a factor of 216 000) dose rate. The observation that saturation occurs in both cases suggests that a multi-track model involving product formation owing to the interaction of two separate tracks is valid for radiation damage in protein crystals. The discrepancy between the optical and EPR saturation dose could be explained by the influence of a number of factors, including sources of error in the measurements or, more probably, the physical conditions under which the different experiments were conducted.

The estimation of the absorbed dose and crystal volume is a source of error for both the UV–visible and EPR measurements. For single-crystal work, the estimation of absorbed dose is now well defined and the limitations of the current *RADDOSE* program have been well documented (Owen *et al.*, 2006 ▶; Paithankar & Garman, 2010 ▶; Paithankar *et al.*, 2009 ▶). In the case of EPR the dose is calculated based on the absorption properties of water. Using crystal properties and X-ray cross-sections at an incident energy of 70 keV, this approximation underestimates the actual dose received by ∼6%, a small error compared with the difference of a factor of 2 in the dose for saturation. For the crystal volume, errors are associated with the accuracy of dimension measurement (±50 µm) and the assumption of a cuboid shape rather than the tetragonal crystal morphology. In the EPR case, the volume measured was in agreement with the volume calculated from the measured radical yield at 20 kGy. Any errors associated with the crystal volume also appear to be small in comparison to the difference in saturation levels and would be expected to be systematic.

The most likely explanation for the differences in the saturation dose is the varying physical conditions of the measurements, *i.e.* temperature, incident X-ray energy and dose rate. Considering temperature first, EPR data are obtained at 4 K to maximize the observed signal to noise, while the optical data were recorded at 100 K. The optical data show that ∼8% of the radicals observed immediately following the pulse have decayed in 5 s (reducing the free-radical concentration observed in the crystal). According to our model, this decay is assigned primarily to reactions of SS-centered radicals with holes and electrons. Reactions of one-electron reduced disulfide bonds with holes yield parent, while reactions with electrons yield product. Over a time scale of seconds, hole/electron transfer may proceed by tunneling or hopping and, given the photon flux density (10^12^ photons s^−1^ in 50 × 50 µm), overlapping tracks are involved. In the EPR measurements at 4 K the tunneling rates would be comparable to those at 100 K, but conversely the hopping rates would effectively be zero. The decay seen in the optical data is not observed within the dose range of the EPR experiment and therefore it is likely that this ∼8% reduction in radical signal at 100 K occurs owing to hopping yielding parent rather than product, *i.e.* a repair process. Saturation would thus occur at a lower dose at 4 K than at 100 K, where hopping is more likely. Experimentally, others have not observed a large difference between data collection at 100 K and lower temperatures. Meents *et al.* (2007 ▶) studied the temperature dependence of radiation-damage rates in holoferritin and insulin crystals, cooling the crystals to 15–90 K with gaseous helium from a liquid-helium cryostat. There was a small positive protective effect on collecting data at 15 K *versus* 90 K. This effect, leading to a decrease in decay of the signal to noise, was greater for holoferritin (23%) than for insulin (6%), possibly owing to the pH dependence of radiation chemistry and the crystallization conditions: pH 11 for insulin and pH 7 for ferritin. However, at 15 K rather than at 4 K as used here, hopping still takes place. It is possible to conduct the optical measurements closer to liquid-helium conditions, but for practical and economic reasons nitrogen gas stream temperature control at 100 K is the standard in the field.

While the type of ice (amorphous, hexagonal or cubic) does not greatly influence the free-radical signal (Johnson & Moulton, 1978 ▶; Bednarek *et al.*, 1998 ▶), the behavior of ice at 4 K and at 100 K might do so. Johnson & Moulton (1978 ▶) noted that the temperature at which ice is irradiated has a significant effect on the free-radical yield of both 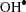 and 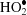 radicals. Annealing and re-cooling of samples showed that radicals formed at low temperature can reversibly evolve at higher temperatures and that samples irradiated at 4 K *versus* 77 K exhibit a higher free-radical yield. EPR peaks in ice for both 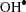 and 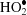 radicals were found at *g* = 2.08 and *g* = 2.05 (Johnson & Moulton, 1978 ▶), which places them outside the maximum *g* value of our simulated disulfide radical, which was 2.02. However, owing to the broad line width of both radicals in question, their presence may have an influence on the integral analysis results of the disulfide radical anion EPR signal. In this context, it is possible that contributions to the EPR signals owing to ice-radical species may result in an overestimation of the free-radical yield of 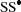 owing to the influence of ice-radical signals. This would not have a deleterious effect on the identification of the EPR disulfide signal, since the *g* values of ice radicals are too large to directly interfere with 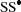 features, but the contribution from the tails of ice-radical signals may increase the signal and thereby cause the EPR 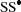 radical yield data to appear to saturate at a lower dose. In comparison, for spectrophoto­metry data, the signal being followed is specific to disulfide radical formation and is not influenced by 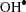 and 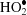 radicals.

The microspectrophotometry data were recorded at an incident X-ray energy of 12.8 keV, whereas the irradiation of the sample studied by EPR was with 70 keV X-rays. X-ray crystallographic studies show that the incident X-ray energy has little overall effect on the rate of radiation damage when the absorbed dose is used as the metric (Gonzalez *et al.*, 1994 ▶; Weiss *et al.*, 2005 ▶) even over as wide a range as 6.5–33 keV (Shimizu *et al.*, 2007 ▶). Other studies covering 3–26 MGy of cumulative absorbed dose on lysozyme indicate a small but consistent energy dependence of the rate of specific damage (Homer *et al.*, 2011 ▶). Homer and coworkers reported lower disulfide-bridge damage for 9 keV incident X-rays than for 14 keV X-rays. While absorbed dose calculations take into account the X-ray cross-sections at different energies, this differential damage rate may be magnified in the 12.8 and 70 keV range used here. Thus, the disulfide bonds in the crystal used in the EPR study, when subjected to 70 keV X-­rays, would be more likely to be damaged at a higher rate than at 12.8 keV and thus saturation would occur at a lower dose point than at the lower incident energies used for the microspectrophotometry and X-ray crystallography measurements. However, this effect, if present, is unlikely to be large enough to explain the difference in saturation values observed in our study.

The variation in the dose rate between the microspectro­photometric and EPR data was significant: up to a 216 000-fold difference. It has been known for some time that free-radical yields in a variety of systems are dependent on the dose rate. An early study utilizing Fricke dosimetry discovered that the production of certain radiation products decreased with increasing intensity of energetic electron pulses (Thomas & Hart, 1962 ▶). This provided early evidence that single-radical chemistry was not an appropriate model for systems with high dose rates. An even earlier study observed a similar effect when tracking the radiation-dependent decolorization of methylene blue (Hutchinson, 1958 ▶). This study concluded that decolorization, which is a radiation-dependent reaction, decreased in samples receiving the same dose at an increased dose rate. This effect can be explained through an increase in the recombination of electrons and electron holes formed in the target: step **2_−_** in Fig. 1 ▶. Higher dose rates would produce holes and electrons in closer proximity, increasing the recombination (or repair) rate and as a result decreasing the stabilization of free radicals compared with lower dose rates. Since the dose rate in microspectrophotometry experiments is over four orders of magnitude higher compared with EPR studies, the dose rate could be a possible explanation for the observed change in disulfide free-radical yield. However, the microspectrophoto­metry data recorded from 1.5 to 270 kGy s^−1^ showed no signs of any systematic dose-rate dependence. Similarly, crystallo­graphically the dose rate for cryocooled samples (unlike the case for room-temperature studies; Owen *et al.*, 2012 ▶) has no clear effect at the macroscopic level, but it has been shown to be a factor in specific radiation damage with increased dose rate, resulting in small but measurable increased damage at radiation-sensitive sites (Se and S atoms; Leiros *et al.*, 2006 ▶). Leiros *et al.* (2006 ▶) studied maltooligosyltrehalose trehalohydrolase and trypsin with tenfold and 24-fold dose-rate differences, respectively. Owen *et al.* (2006 ▶) reported a similar effect, observing a 10% lifetime decrease on a tenfold increase in the dose rate for apoferritin when monitoring global damage in the form of the loss of diffraction intensity. The addition of metal atoms within the protein (iron in holoferritin) produced a more pronounced effect, yielding a 10% lifetime decrease on a threefold increase in dose rate. This appears to be the converse of our observation of a decreased saturation dose with a lower dose rate (EPR measurements), although the effects are small compared with the role played by the total absorbed dose. We note that the difference in dose rates used in our experiments is so large that caution must be taken in any comparison.

While it is technically possible to increase the dose rate for the EPR studies, it is practically unfeasible given the scale of the EPR equipment and laboratory X-ray sources with which it could be coupled. Reducing the dose rate in the microspectrophotometry experiment is also possible through beam attenuation, but again faces a practical limit in terms of the longer beamtime thus required. Additional studies will be required on a laboratory source with online microspectro­photometry capability. It is possible that the combination of dose rate and temperature may play a role in explaining the differences.

In addition to temperature, energy and dose rate, another influence on radiation chemistry is the oxygen or O_2_ effect. Oxygen can both sensitize and protect molecules from free-radical damage, depending on the environment and the specific type of damage considered. Chan & Bielski (1973 ▶) measured the decay rate of the absorption peak owing to one-electron reduced disulfide as a function of molecular oxygen concentration. They found that the decay rates increased from 3.22 × 10^4^ to 1.63 × 10^5^ s^−1^ with an oxygen concentration increasing from 2.08 × 10^−5^ to 1.25 × 10^−4^ *M*, respectively. This led to the conclusion that O_2_ oxidizes disulfide radical anions, resulting in lower yields. This was also observed by Barton & Packer (1970 ▶), who explored the pH dependence of the O_2_ effect. In EPR studies, samples are held under vacuum in an environment where oxygen and nitrogen are excluded to prevent the formation of nitrogen or oxygen ice at liquid-helium temperatures. The microspectrophoto­metry experiments are performed on lysozyme crystals within a nitrogen stream, where there is more possibility of access to oxygen. It is likely that this effect only has a marginal influence, since the permeability of oxygen will be limited and the concentration in the stream, if any, will be low.

The saturation level determined at different dose rates by microspectrophotometry averages at 521 kGy with a standard deviation of 154 kGy for the single-exponential fit and 771 kGy with a standard deviation of 267 kGy for the double-­exponential fit. The microspectrophotometry data are recorded at an orientation in which the cleanest signal is seen. This can depend on the amount of cryobuffer surrounding the crystal, the position of the loop, the crystal morphology and the crystal thickness *etc.*, all of which add experimental variability to the measurement. It may also offer some explanation for the differing saturation level between the microspectrophoto­metry data and the EPR data, in that the absolute free-radical yield for the EPR is based on the data set from crystal 1 only.

The saturation level determined by EPR, especially given the above considerations, is compatible with the range of levels observed in the microspectrophotometry measurements. This is all the more remarkable considering the difference in X-ray flux (and thus dose rates) associated with the two experiments. Furthermore, our model fits well across a range of X-ray doses, explaining the lysozyme data from 5 kGy to 1.05 MGy (EPR and crystallographic studies) and the microspectrophotometry data up to ∼5 MGy.

Other studies of disulfide damage have shown disulfide scission, in particular that of Petrova *et al.* (2010 ▶) on elastase, which spanned doses of 1.2–30 MGy. We differ from Petrova and coworkers in the interpretation of the processes underlying their observations, but only in terms of the development of our multi-track model, which was not yet available at the time of the elastase study. At even the smallest absorbed dose in our range (5 kGy), the EPR measurements reported here indicate complete dose saturation of one-electron reduced disulfide bonds within the protein. In addition, our model predicts that the initial reduction of disulfide bridges would not result in the scission of the bond. The disulfide scission observed by Petrova *et al.* (2010 ▶) is likely to be owing to two one-electron reduction events at the disulfide bonds of elastase. Unlike the elastase study, in lysozyme no large-scale rigid-body structural changes or significant elongation of disulfide bonds are observed. This is not surprising given that the dose range of our studies ends before that of the first data set of the elastase study starts, and our results are not inconsistent with their observations. For elastase, the formation of alternate conformations for cysteines that are water-accessible is seen. At the absorbed doses of our study, only Cys94 of the Cys76–Cys94 disulfide bond forms an alternate conformation. Of the two cysteines making up this bond, Cys94 has the lower water-accessibility but Cys76 does not show evidence of developing different conformations. We speculate that the production of a cysteine rotamer is an indication that cysteine is the major and perhaps the only product (*P* in equation 1). The disulfide bond with the highest solvent accessibility, Cys6–­Cys127, shows no evidence of developing any alternate conformations in our study. It would appear that structural perturbation owing to ongoing radiation chemistry is both dose- and environment-specific.

An important aspect of the experimental results presented here is the observation of radical formation even at the lowest doses used of 5 kGy. X-ray crystallography-based radiation-damage studies are limited in that to observe structural changes the starting structure has to be determined through the very mechanism that is being investigated. Our results indicate that even at the lowest doses used for structural investigations, disulfide bonds are already becoming radicalized. Extra electron density is present, which if not taken into account could give misleading results when trying to quantitate damage observed from difference-map techniques. Practically, there are few ways to avoid this. One solution may be to use neutron diffraction to provide an unbiased baseline structure, but no radiation-damage studies have made use of this approach to date. Our model allows us to understand the nature of disulfide-bond loss in lysozyme crystals and can potentially be extended to predict the lability of each amino-acid side chain within a protein. More work is required to empirically test this protein-damage model, in which other local factors should also be considered, such as solvent accessibility and the proximity of other amino-acid side chains, all of which are a consequence of secondary and tertiary protein structure.

## 6. Conclusion   

Understanding radical destruction as well as formation is key to understanding the radiation-induced changes that impact X-ray diffraction data. A multi-track model involving both formation and destruction has been shown here to be valid in explaining X-ray-induced disulfide-bond damage, since it fits UV–Vis, EPR and both low-dose and high-dose crystallo­graphic data. Multi-track considerations offer the first step in a comprehensive model of radiation damage that could potentially lead to a combined computational and experimental approach to identify when damage is likely to be present, to quantitate it and to provide the ability to recover the native unperturbed structure. Intriguingly, a successful model would not only allow treatment of new structural information but, in cases where the absorbed dose has been recorded, would also allow identification and potential remediation of previously deposited structural data.

## Supplementary Material

PDB reference: lysozyme, 4h8x

PDB reference: 4h8y

PDB reference: 4h8z

PDB reference: 4h90

PDB reference: 4h91

PDB reference: 4h92

PDB reference: 4h93

PDB reference: 4h94

PDB reference: 4h9a

PDB reference: 4h9b

PDB reference: 4h9c

PDB reference: 4h9e

PDB reference: 4h9f

PDB reference: 4h9h

PDB reference: 4h9i

Zip file of PDB depositions. DOI: 10.1107/S0907444913022117/kw5071sup1.zip

Supplementary material. DOI: 10.1107/S0907444913022117/kw5071sup2.pdf

## Figures and Tables

**Table 1:** Crystal dimensions and dose points used for the EPR measurements

	Dimensions (mm)	Volume† (mm^3^)	Mass(g)	Dose points for EPR measurements (kGy)
Crystal 1	0.60 0.50 0.40	0.12	208	5, 10, 20, 40, 60, 100, 150
Crystal 2	0.50 0.50 0.25	0.06	135‡	10, 20, 40, 60, 100
Crystal 3	0.50 0.50 0.40	0.10	185‡	20, 40, 100, 200, 300, 400, 500

† Volume is approximate and was calculated by assuming a cuboid which does not take into account crystal shape.

‡ Masses were calculated based on the measured radical yield at a dose of 20kGy as detailed in [Sec sec3]3.

**Table 2:** Saturating dose, *D*_90_, for each of the eight lysozyme crystals obtained using both a single- and a double-exponential fit to the data Note that crystals 1 and 2 were measured on different experimental runs and, although in both the beam was not attenuated, they were subjected to slightly different incident fluxes. Crystals 28 were measured on the same experimental run.

Crystal	Attenuation (%)	Flux (photonss^1^)	Dose rate (kGys^1^)	*D*_90_, single (kGy)	*D*_90_, double (kGy)
1	0	1.51 10^12^	270	772	921
2	0	1.34 10^12^	240	451	557
3	48.0	6.78 10^11^	121	465	376
4	73.0	3.45 10^11^	62	711	928
5	90.0	1.08 10^11^	19	289	533
6	96.0	4.67 10^10^	8.4	541	1183
7	98.2	1.99 10^10^	3.5	482	819
8	99.2	8.58 10^9^	1.5	461	716
Average				521	771
Standard deviation				154	267

**Table 3:** Crystallographic data and structural refinement statistics for a lysozyme crystal from which structural X-ray data were collected (complete statistics for each data set are available in the Supplementary Material) The absorbed dose for each data set was 0.07MGy, with 15 data sets giving a total absorbed dose of 1.05MGy.

	First data set	Last data set	Mean	Standard deviation
Data-collection statistics				
Unit-cell parameters				
*a* = *b* ()	78.77	78.77	78.76	0.006
*c* ()	36.86	36.87	37.39	0.004
Wilson *B* factor (^2^)	10.7	11.3	11.1	0.16
Structural statistics				
*R*_work_/*R*_free_ (%)	0.19/0.20	0.19/0.20	0.19/0.20	0/0
SS bond length ()				
Cys6Cys127	2.04	2.05	2.04	0.0051
Cys30Cys115	2.05	2.09	2.09	0.0139
Cys64Cys80	2.04	2.05	2.04	0.0046
Cys76Cys94	2.02	2.04	2.03	0.0077

**Table 4:** Solvent accessibilities for the disulfide residues in lysozyme obtained using the program *AREAIMOL* Note that any conclusions have to be tempered by the limited precision of such calculations (Novotny *et al.*, 2007 ▶).

Bond	Residue	Solvent accessibility (^2^)
Cys6Cys127	Cys6	43
	Cys127	21
Cys30Cys115	Cys30	0
	Cys115	0
Cys64Cys80	Cys64	0
	Cys80	1
Cys76Cys94	Cys76	20
	Cys94	2

## Footnotes

1 Supplementary material has been deposited in the IUCr electronic archive (Reference: KW5071). Services for accessing this material are described at the back of the journal.
