# Supplementary material for: Insights into the mechanism of X-ray-induced disulfide-bond cleavage in lysozyme crystals based on EPR, optical absorption and X-ray diffraction studies
Source: Acta Crystallogr D Biol Crystallogr. 2013 Nov 19;69(Pt 12):2381–94. doi: 10.1107/S0907444913022117 (PMC3852651; doi:10.1107/S0907444913022117)
Supplement: Supplementary file 2 [file d-69-02381-sup2.pdf]

Insights into the mechanism of X-ray induced disulfide bond cleavage in lysozyme crystals based on EPR, optical absorption, and X-ray diffraction studies

Kristin A. Sutton[a], Paul Black[b+], Kermit R. Mercer[b], Elspeth F. Garman[c], Robin L. Owen[d], Edward H. Snell[ae]* and William A. Bernhard[b]

[a]Hauptman Woodward Medical Research Institute, 700 Ellicott St., Buffalo, NY, 14086, United States, [b]University of Rochester Medical Center, Rochester, NY, 14642, United States, [c]Laboratory of Molecular Biophysics, Department of Biochemistry, University of Oxford, South Parks Road, Oxford, Oxfordshire, OX1 3QU, United Kingdom, [d]Diamond Light Source, Harwell Science and Innovation Campus, Didcot, Oxfordshire, OX1 3QU, United Kingdom, and [e]Department of Structural Biology, SUNY Buffalo Medical School, 700 Ellicott St., Buffalo, NY, 14203, United States

+Current address: Wake Forest Baptist Health, Medical Center Blvd, Winston-Salem, NC, 27127, United States.

Supplementary Material

Table S1. Crystallographic statistics

Dataset #	1	2	3	4	5	6	7	8
PDB ID	4H8X	4H8Y	4H8Z	4H90	4H91	4H92	4H93	4H94
Cumulative Dose (MGy)	0.07	0.14	0.21	0.28	0.37	0.42	0.49	0.56
Space group	$P4_32_12$							
Unit cell								
a=b (Å)	78.76	78.76	78.76	78.76	78.76	78.76	78.76	78.76
c (Å)	38.86	38.86	38.86	38.86	38.86	38.86	38.86	38.86
Resolution (Å)	29.91-1.20 (1.23-1.20)							
Unique refs.	36614	36582	36582	36624	36589	36591	36593	36614
Observed refs.	163785	163674	163670	163684	163577	163579	163581	163640
Multiplicity	4.5	4.5	4.5	4.5	4.5	4.5	4.5	4.5
Completeness (%)	97.53 (94)	99.33 (100)	99.33 (100)	99.34 (100)	99.34 (100)	99.34 (100)	99.34 (100)	99.33 (100)
$<I/\sigma(I)>$	45.1 (5.0)	43.2 (5.4)	43.2 (5.4)	45.7 (5.3)	44.1 (5.3)	43.6 (5.2)	44.5 (5)	44.7 (5)
R_{merge} (%)	3 (30)	3 (28)	3 (28)	3 (28)	3 (28)	3 (29)	3 (29)	3 (30)
(%)								
Dataset #	9	10	11	12	13	14	15	
PDB ID	4H9A	4H9B	4H9C	4H9E	4H9F	4H9H	4H9I	
Cumulative Dose (MGy)	0.63	0.70	0.77	0.84	0.91	0.98	1.05	
Space group	$P4_32_12$							
Unit cell								
a=b (Å)	78.76	78.76	78.76	78.76	78.77	78.77	78.77	
c (Å)	38.86	38.86	38.86	38.86	38.86	38.87	38.87	
Resolution (Å)	26.91-1.2 (1.23-1.2)							

Unique refs.	36644	36643	36644	36646	36603	36618	36619	
Observed refs.	163771	163782	163763	163823	163625	163717	163715	
Multiplicity	4.5	4.5	4.5	4.5	4.5	4.5	4.5	
Completeness (%)	99.33 (100)	99.33 (100)	99.34 (100)	99.34 (100)	99.34 (100)	99.34 (100)	99.35 (100)	
$\langle I/\sigma(I)\rangle$	44.1 (4.9)	44.5 (4.9)	44.4 (4.9)	44.4 (4.8)	44.5 (4.8)	44.6 (4.7)	44.6 (4.7)	
R_{merge}(%)	3 (30)	3 (30)	3 (31)	3 (30)	3 (31)	3 (31)	3 (32)	

Table S2. Model generation and refinement statistics.

Dataset #	1	2	3	4	5	6	7	8
PDB ID	4H8X	4H8Y	4H8Z	4H90	4H91	4H92	4H93	4H94
Cumulative Dose (MGy)	0.07	0.14	0.21	0.28	0.35	0.42	0.49	0.56
Solvent (%)	38.35	38.31	38.31	38.32	38.32	38.33	38.33	38.33
R_{work}	0.20	0.20	0.19	0.19	0.20	0.19	0.20	0.20
R_{free}	0.21	0.21	0.21	0.21	0.21	0.21	0.21	0.21
No. residues	129							
No. waters	102	107	117	123	102	116	115	113
Ramachandran region (favored/allowed/outliers) (%)	97.69/ 2.31/ 0	98.46/ 1.54/ 0	97.69/ 2.31/ 0	97.69/ 2.31/ 0	97.69/ 2.31/ 0	97.69/ 2.31/ 0	97.69/ 2.31/ 0	97.69/ 2.31/ 0
RMSD bond length (Å)	0.005	0.005	0.005	0.005	0.005	0.005	0.007	0.005
RMSD bond angle (°)	0.98	1.04	1.04	1.04	1.04	1.04	1.25	1.04
Average B factor (Å^2)	13.26	13.23	13.21	13.36	13.36	13.32	13.48	13.16
Molprobity clash score	2.4	4.9	2.4	2.4	3.9	2.4	2.0	2.4
Dataset #	9	10	11	12	13	14	15	
PDB ID	4H9A	4H9B	4H9C	4H9E	4H9F	4H9H	4H9I	
Cumulative Dose (MGy)	0.63	0.7	0.77	0.84	0.91	0.98	1.05	
Solvent (%)	38.34	38.35	38.35	38.35	38.36	38.36	38.36	
R_{work}	0.20	0.20	0.20	0.20	0.20	0.20	0.20	
R_{free}	0.21	0.21	0.21	0.21	0.21	0.21	0.21	
No. residues	129							
No. waters	110	101	104	108	102	112	109	
Ramachandran (favored/allowed/outliers) (%)	97.69/ 2.31/ 0	97.69/ 2.31/ 0	97.69/ 2.31/ 0	97.69/ 2.31/ 0	97.69/ 2.31/ 0	97.69/ 2.31/ 0	97.69/ 2.31/ 0	
RMSD bond length (Å)	0.006	0.005	0.005	0.006	0.006	0.006	0.006	
RMSD bond angle (°)	1.03	1.03	1.03	1.04	1.04	1.06	1.05	
Average B factor (Å^2)	13.31	13.28	13.2	13.47	13.54	13.88	13.73	
Molprobity clash score	3.4	2.9	2.9	3.4	2.9	3.4	3.4	

Table S3. Table 2 from the paper with saturating dose, D_{90}, for each of the eight lysozyme crystals measured, extracted from both a single and double exponential fit to the data. The parameters fitted to

the dose response curves are specifically listed. Note that crystals 1 and 2 were measured on different experimental runs and although in both the beam was not attenuated, they were subjected to slightly different incident fluxes. Crystals 2-8 were measured on the same experimental run.

Crystal	Attenuation (%)	Flux (ph s^{-1})	Dose rate (kGy s^{-1})	d_1	d_2	B_1	B_2	D_{90} single (kGy)	D_{90} double (kGy)
1	0	1.51×10^{12}	270	1233.6	127.3	-0.1367	-0.0738	772	921
2	0	1.34×10^{12}	240	436.9	86.9	-0.1215	-0.3646	451	557
3	48.0	6.78×10^{11}	121	227.8	36.9	-0.0578	-0.4942	465	376
4	73.0	3.45×10^{11}	62	723.4	106.2	-0.1231	-0.0802	711	928
5	90.0	1.08×10^{11}	19	286.2	52.4	-0.1367	-0.0738	289	533
6	96.0	4.67×10^{10}	8.4	976.2	116.5	-0.0852	-0.1581	541	1183
7	98.2	1.99×10^{10}	3.5	570.9	67.5	-0.0747	-0.0979	482	819
8	99.2	8.58×10^{9}	1.5	521.3	67.0	-0.1035	-0.0898	461	716
Average								521	771
Standard deviation								154	267

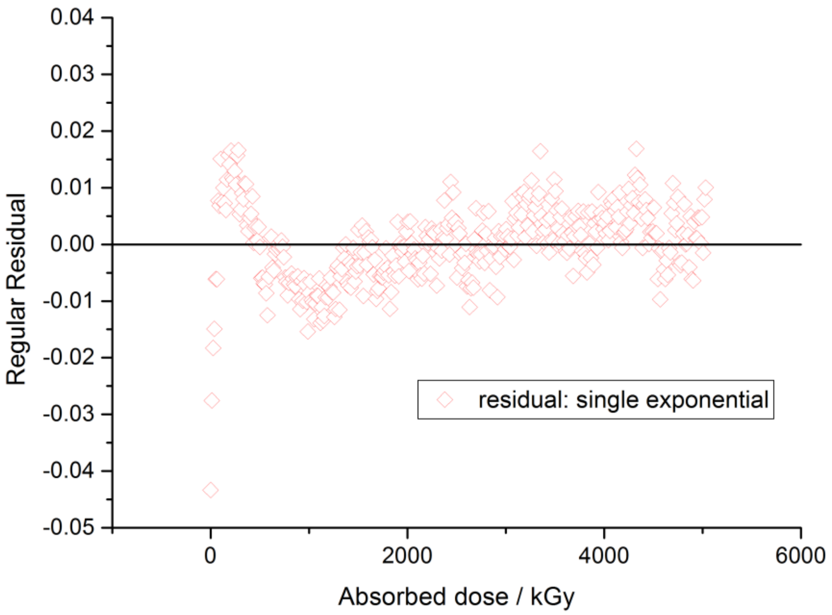

(a)

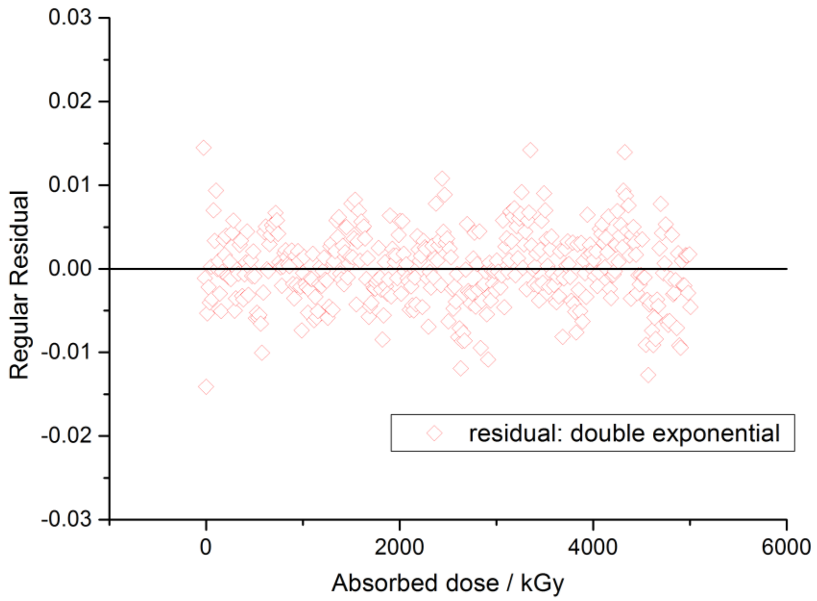

(b)

Figure S1. Residuals for the (a) single exponential fit and (b) the double exponential fit to the data shown in Figure 2(b) of the main text.
